# The 2nd Next Generation Conversation: Albany @ Ruston

**DOI:** 10.1007/s12551-026-01472-5

**Published:** 2026-10-08

**Authors:** Thomas C. Bishop, Aditya Mittal, Freddie R. Salsbury, Kelly M. Thayer, Robert T. Young

**Affiliations:** 1 Departments of Chemistry and Physics, Louisiana Tech University, Ruston, LA 71272 USA; 2 Kusuma School of Biological Sciences and Supercomputing Facility for Bioinformatics & Computational Biology, Indian Institute of Technology Delhi, Hauz Khas, New Delhi 110016 India; 3 Department of Physics, Wake Forest University, Winston-Salem, NC 27109 USA; 4 College of Integrative Sciences, Departments of Chemistry and Computer Science, Wesleyan University, Middletown, CT 06457 USA

**Keywords:** Commentary, Albany Conversations, Next Gen Conversations, Conference

## Abstract

This commentary is a report on the 2nd Next Gen. Conversation: Albany @ Ruston 2026 (the 22nd Albany Conversation) held on the campus of Louisiana Tech University June 9–13, 2026. The 3rd Next Gen. Conversation will be held at Louisiana Tech University June 13–17, 2028. Future conversations will begin on the 2nd Tuesday of June every other year.

## Commentary

The 2nd Next Gen. Conversation: Albany at Ruston (22nd Albany Conversation) was held on the campus of Louisiana Tech University on June 9–13, 2026. It was held in the Prescott Memorial Library overlooking Louisiana Tech’s North Quad and the “Lady in the Mist”.

The Next Gen. Conversations are a continuation of the Albany Conversations in Biomolecular Stereo-Dynamics started in 1979 by the late Prof. Ramaswamy H. Sarma at the State University of New York in Albany. Ruston, like Albany, is within close proximity to large cities with major research institutions yet is itself sufficiently isolated to provide a distraction-free venue that supports scientific conversations and the development of collaborative research networks and ideas (Bishop, Thayer, Young 2024) . The 1st and 2nd Next Gen. Conversations at Louisiana Tech successfully captured the unique, collaborative spirit of the original Albany Conversations. Support was provided by the National Science Foundation under Award No. NSF 2412981 with additional support for international visitors provided by Louisiana Tech President Jim Henderson. The Next Gen. Conversations will advance the legacy of Sarma’s Albany Conversations.

**Fig. 1:**
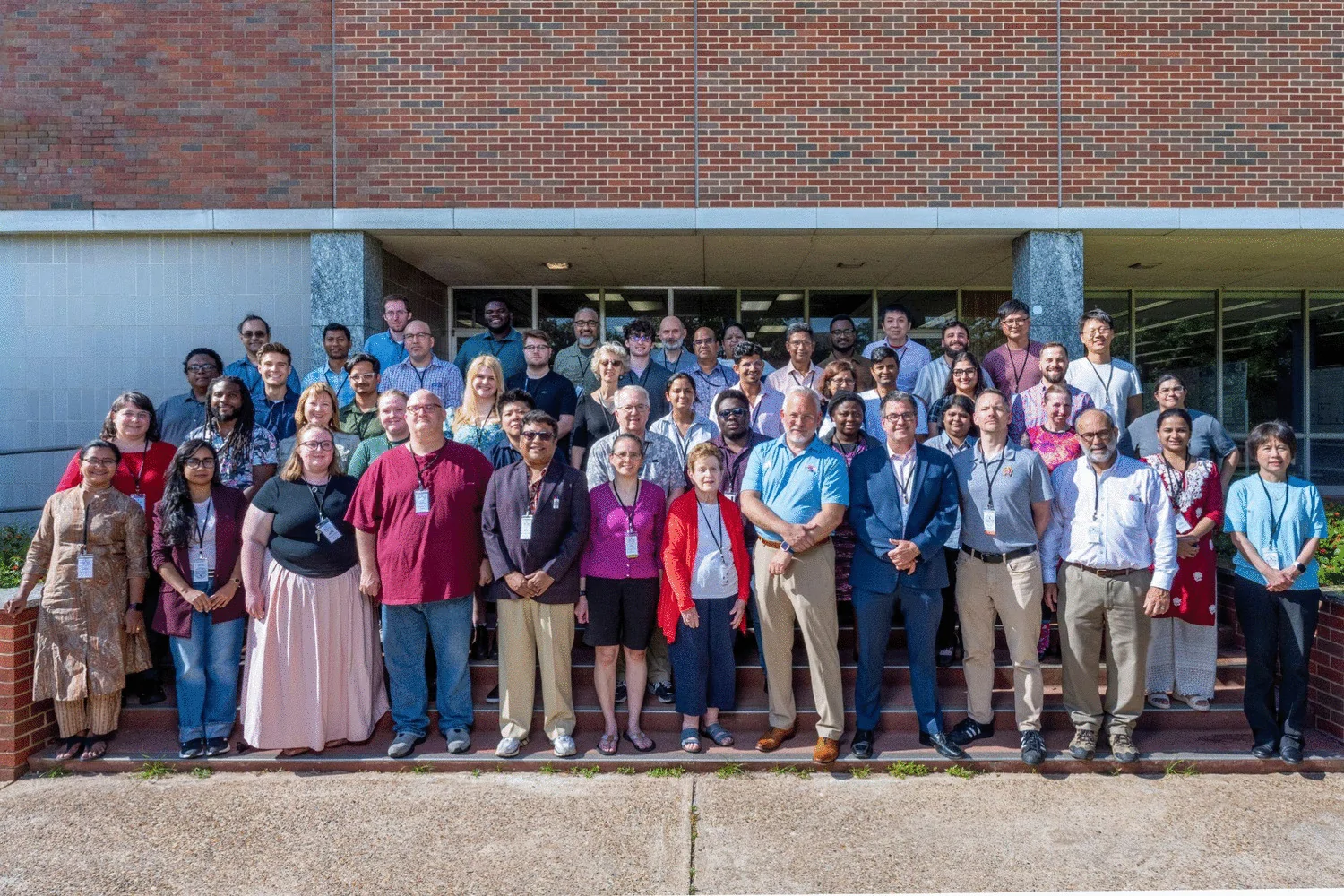
Conversation participants gather on the front porch of Prescott Memorial Library at Louisiana Tech University to start off the 2026 meeting. Photo courtesy of University Communications

The 2nd Next Gen. Conversation had about 60 attendees (Fig. 1) who gathered to discuss the full S.E.T. (Simulation, Experiment, and Theoretical) of approaches to the study of biomolecular physics and informatics. The cohort of researchers was diverse and ranged from undergraduate students to world-renowned experts from primary undergraduate to research-intensive universities located in rural and major metropolitan areas across the globe. Despite serious travel-associated challenges due to the tense global socio-political situations, the 2nd Next Gen. Conversation almost doubled the total participation compared to the 1st Next Gen. Conversation and saw an increase in the number of posters presented from 5 to 27. Participants were from the USA, UK, India, and Israel, with some (last-minute) cancellations from Russia, Armenia, and Uruguay due to travel challenges. From within the USA, participants hailed from 14 states (AL, CA, CT, IL, LA, MD, MS, NC, NJ, NY, OH, SC, TN, TX). This made the 2nd Next Gen. Conversation a unique state-of-the-art scientific gathering in the rural South, specifically highlighting the role of biophysics as an important inter- and multi-disciplinary field of research in the modern world. The topics of conversation included computational-, structural-, and systems biology, biological dynamics, large data and data analytics pertaining to structure–function relationships in DNA, proteins and whole cells. The complete Book of Abstracts is available electronically as a special issue of the Journal of Biomolecular Structure and Dynamics (Young, Thayer, Salsbury, Mittal & Bishop 2026).

The 2nd Conversation was initiated by Prof. B. Ramu Ramachandran, the Interim Vice President for Research at Louisiana Tech, with a warm welcome and acknowledgments of key efforts and support. This year’s Conversation overlapped with Tech’s 2026 first freshmen orientation session, yet President Henderson still managed to join us after Remo Rohs’ keynote address for a crawfish boil on the library’s “Front Porch.”

The Conversation organizers, Tom Bishop of Louisiana Tech University, Kelly M. Thayer of Wesleyan University, Aditya Mittal of IIT Delhi, Freddie Salsbury of Wake Forest University, and Robert Young of Louisiana Tech University, began the sessions with their “Conversation Starters” and a solemn recognition of the legacy left by Prof. Ramaswamy H. Sarma, fondly called Rama/Sarma/Prof. Sarma/Prof. Rama/Ramaswamy, who passed away on November 20, 2025. Following the Albany Conversation traditions, this session also honored distinguished alumni who passed away between the 1st and 2nd Next Gen. Conversations with fond memories: Richard E. Dickerson (University of California, Los Angeles), Neville R. Kallenbach (New York University), Martin Karplus (Harvard University), Richard Lavery (Centre National de la Recherche Scientifique (CNRS), France), George A. Petersson (Wesleyan University), Wolfram Saenger (Freie Universität Berlin), Heinz Sklenar (Max Delbrück Center for Molecular Medicine, Berlin), and Hamilton O. Smith (Johns Hopkins University). It was noted that attendance at the 2nd Next Gen. Conversation included attendees from the initial Albany Conversation, their third and fourth generation academic progeny along with several first-timers. A plenary talk was delivered by the senior-most veteran, Wilma Olson, while the keynote was from Remo Rohs, a 25-year Conversation veteran who gave his first talk and poster at these meetings. He now heads the Department of Quantitative and Computational Biology at USC, which has several NAS fellows and a Nobel Laureate.

The first set of scientific talks was delivered by Lynn Zechiedrich, Haley Johnson (both from Baylor College of Medicine), and Rebecca Giorno-McConnell (Louisiana Tech). Discussions ranging from the shapes of DNA and conformations for the genome to survival studies of spores in space exploration set the right tone for engaging intellectual conversations for the duration of the meeting. Afternoon and evening sessions featured Binay Panda (JNU, India), Suma Mohan (Sastra U, India), Shuaihua Gao (USC, USA), Viji Mahadevan (IBAB, India) and Jinsen Li (USC, USA).

**Fig. 2:**
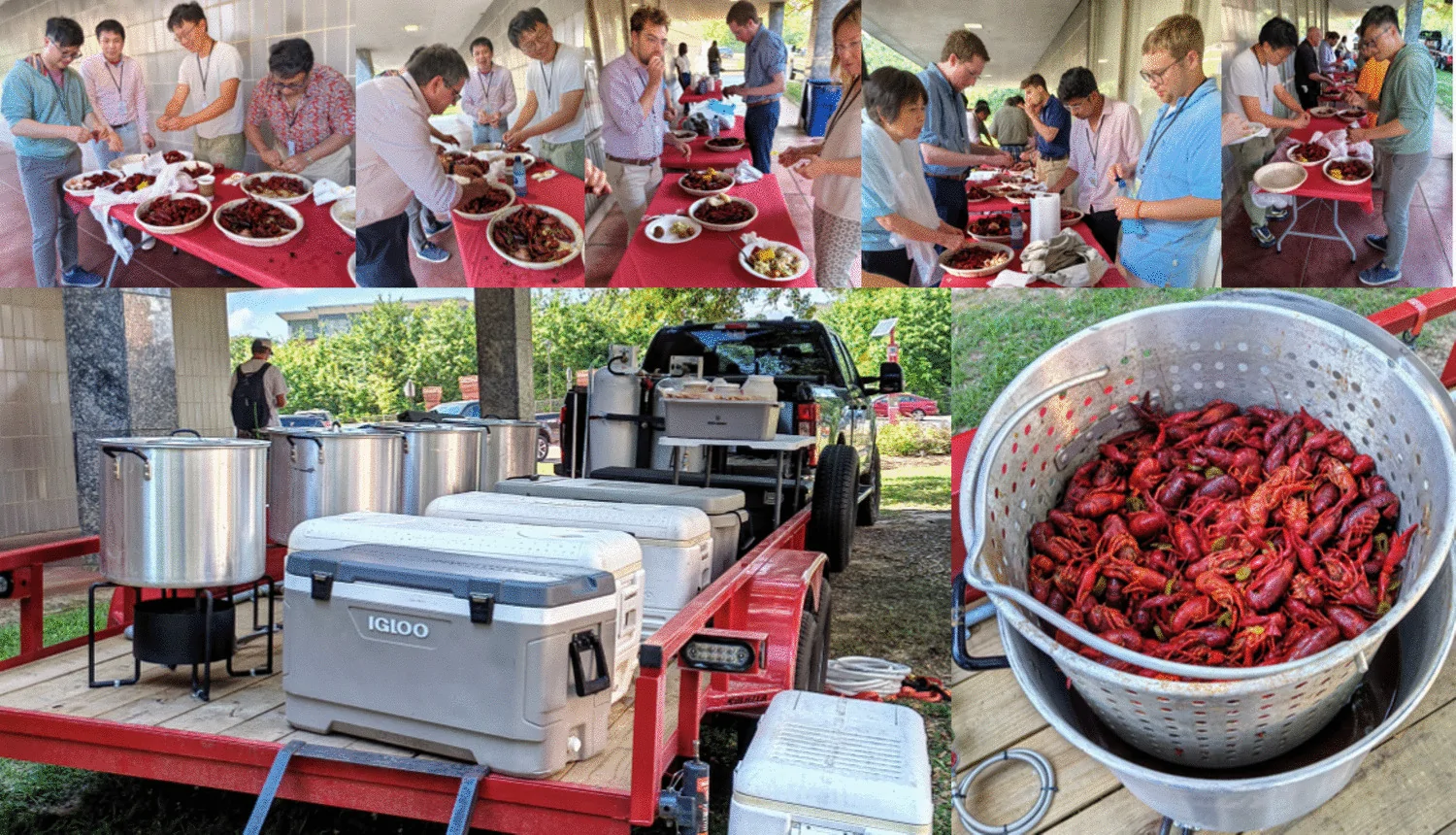
Crawfish boil. After the keynote address delivered by Remo Rohs, participants partook of a favorite local custom, an onsite crawfish boil and outdoor summer dining adjacent to the library. Comradery among participants was fueled by this tactile eating experience of tantalizing local cuisine. Photos courtesy of Lindsay Zack

The second day began with another first for the Next Gen. Conversations: Freddie Salsbury (Wake Forest U, USA) hosted an editorial board meeting for the Journal of Biomolecular Structure and Dynamics (JBSD) as its Editor-in-Chief, with virtual participation of Editorial Advisory Board (EAB) members from Brazil, India, Mexico, Russia, South Africa, Turkey, the UK, and the USA (CT, FL, KY) as well as in-person EAB members. This was followed by engaging talks from Tsu-Pei Chiu (USC, USA), Sarah Harris (U Leeds, UK), Yongmei Wang (U Memphis, USA), Laura Finzi (Clemson U, USA), and a captivating keynote address by Remo Rohs (USC, USA). Rohs presented on the timely topic of computational structural biology in the era of artificial intelligence. The day ended with a crawfish boil at the library’s front porch and its adjacent garden (Fig. 2).

The third day began with Manju Hingorani (NSF, USA) discussing research funding opportunities. This was followed by thought-provoking talks from Ikponwmosa Obaseki (Miami U Ohio, USA), Surabhi Johari (IMS, India), James Gomes (IIT Delhi, India), Hannah Popoola (Miami U Ohio, USA), Tapan K. Nayak (IIT Delhi, India), and Haim Rozenberg (Weizmann Institute, Israel). These were followed by five short talks from amongst the student (undergraduate and graduate) researchers who were recognized for their outstanding poster presentations. Four graduate students were also recognized for their outstanding talk or poster by JBSD. The day ended with a wonderful plenary from the seniormost participant, but ever youthful, Wilma Olson (Rutgers U, USA).

**Fig. 3:**
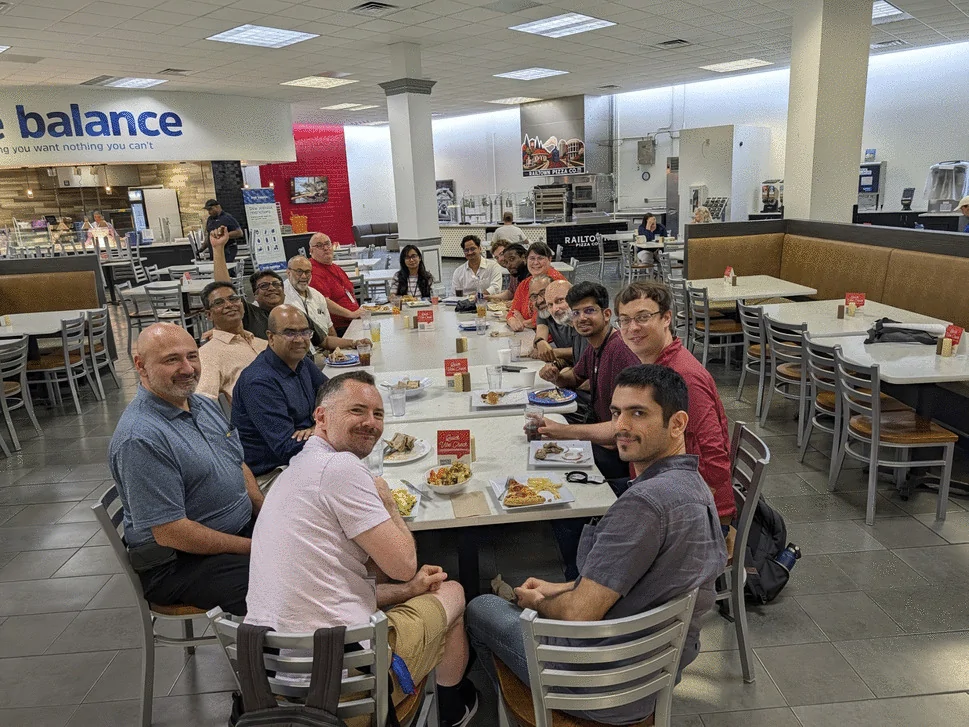
Lunch at Tech Table. The Conversations provide a seat at the table for participants at all levels to engage informally in scientific discussions and collegial comradery over meals served at the LA Tech’s campus cafeteria. Photo courtesy of Tech Table

In terms of impact, the 2nd Next Gen. Conversation did achieve its goals of (a) carrying forward the legacy of “Uncle Rama” and his Albany Conversations and (b) highlighting the important role of biophysical research in general. Notably, some labs arrived with almost their entire group. For them, the Conversation also served as a shared/team building experience. For others, especially foreign nationals, it was solo travel in challenging times to quench their scientific thirst. For all, the Conversation served the purposes of reconnecting with colleagues from their earlier studies/careers, establishing new collaborations and exploring career development opportunities (Fig. 3).

The Conversations will continue to be an international biennial meeting series focused on recent advances in biomolecular structure, dynamics and function that embraces simulation, theory and experiment. The 3rd Next Gen. Conversation: Albany at Ruston will be a Tuesday to Saturday meeting on the campus of Louisiana Tech University June 13–17, 2028. We are actively seeking to expand the size and scope of the Conversations. If you are interested in organizing a session, please contact the conference organizers at albany@latech.edu. Visit the conference website for additional updates and information https://dna.engr.latech.edu/NextGen/.

## Data Availability

The conference email address is albany@latech.edu and the conference web address is https://dna.engr.latech.edu/NextGen/.

## Data vailability

No datasets were generated or analysed during the current study.
